# Successful Control of Ebola Virus Disease: Analysis of Service Based Data from Rural Sierra Leone

**DOI:** 10.1371/journal.pntd.0004498

**Published:** 2016-03-09

**Authors:** Kamalini Lokuge, Grazia Caleo, Jane Greig, Jennifer Duncombe, Nicholas McWilliam, James Squire, Manjo Lamin, Emily Veltus, Anja Wolz, Gary Kobinger, Marc-Antoine de la Vega, Osman Gbabai, Sao Nabieu, Mohammed Lamin, Ronald Kremer, Kostas Danis, Emily Banks, Kathryn Glass

**Affiliations:** 1 Manson Unit, Médecins Sans Frontières, London, United Kingdom; 2 National Centre for Epidemiology and Population Health, Research School of Population Health, Australian National University, Canberra, Australia; 3 Médecins Sans Frontières, Kailahun, Sierra Leone; 4 District Health Management Team, Ministry of Health and Sanitation, Kailahun, Sierra Leone; 5 Operational Centre Geneva, Médecins Sans Frontières, Geneva, Switzerland; 6 National Microbiology Laboratory, Public Health Agency of Canada, University of Manitoba, Winnipeg, Canada; 7 Operational Centre Amsterdam, Médecins Sans Frontières, Amsterdam, The Netherlands; 8 European Programme for Intervention Epidemiology Training (EPIET), European Centre for Disease Prevention and Control, (ECDC), Stockholm, Sweden; 9 Institut de Veille Sanitaire, Paris, France; Common Heritage Foundation, NIGERIA

## Abstract

**Introduction:**

The scale and geographical distribution of the current outbreak in West Africa raised doubts as to the effectiveness of established methods of control. Ebola Virus Disease (EVD) was first detected in Sierra Leone in May 2014 in Kailahun district. Despite high case numbers elsewhere in the country, transmission was eliminated in the district by December 2014. We describe interventions underpinning successful EVD control in Kailahun and implications for EVD control in other areas.

**Methods:**

Internal service data and published reports from response agencies were analysed to describe the structure and type of response activities, EVD case numbers and epidemic characteristics. This included daily national situation reports and District-level data and reports of the Sierra Leone Ministry of Health and Sanitation, and Médecins Sans Frontières (MSF) patient data and internal epidemiological reports. We used EVD case definitions provided by the World Health Organisation over the course of the outbreak. Characteristics assessed included level of response activities and epidemiological features such as reported exposure (funeral-related or not), time interval between onset of illness and admission to the EVD Management Centre (EMC), work-related exposures (health worker or not) and mortality. We compared these characteristics between two time periods—June to July (the early period of response), and August to December (when coverage and quality of response had improved). A stochastic model was used to predict case numbers per generation with different numbers of beds and a varying percentage of community cases detected.

**Results:**

There were 652 probable/confirmed EVD cases from June-December 2014 in Kailahun. An EMC providing patient care opened in June. By August 2014 an integrated detection, treatment, and prevention strategy was in place across the district catchment zone. From June-July to August-December 2014 surveillance and contact tracing staff increased from 1.0 to 8.8 per confirmed EVD case, EMC capacity increased from 32 to 100 beds, the number of burial teams doubled, and health promotion activities increased in coverage. These improvements in response were associated with the following changes between the same periods: the proportion of confirmed/probable cases admitted to the EMC increased from 35% to 83% (**χ**^**2**^ p-value<0·001), the proportion of confirmed patients admitted to the EMC <3 days of symptom onset increased from 19% to 37% (**χ**^**2**^ p-value <0·001), and reported funeral contact in those admitted decreased from 33% to 16% (**χ**^**2**^ p-value <0·001). Mathematical modelling confirmed the importance of both patient management capacity and surveillance and contact tracing for EVD control.

**Discussion:**

Our findings demonstrate that control of EVD can be achieved using established interventions based on identification and appropriate management of those who are at risk of and develop EVD, including in the context of ongoing transmission in surrounding regions. Key attributes in achieving control were sufficient patient care capacity (including admission to specialist facilities of suspect and probable cases for assessment), integrated with adequate staffing and resourcing of community-based case detection and prevention activities. The response structure and coverage targets we present are of value in informing effective control in current and future EVD outbreaks.

## Author Summary

Ebola Virus Disease (EVD) is a severe illness that is usually spread from person to person through caring for someone who is sick, or if they die, contact with their body during their funeral. The recent EVD outbreak in West Africa caused illness and death in many thousands in Guinea, Sierra Leone and Liberia. It has been the largest and most difficult to control of any EVD outbreak in history, and this led to doubts as to the effectiveness of established control measures. Our study describes the successful control of EVD in a rural district of Sierra Leone. As in previous outbreaks, we found that control was achieved by working with communities to identify people who may have been exposed to EVD; if they then became sick, their early admission for testing and care to specialised centres that have equipment and procedures to prevent EVD passing on to staff or other patients, and safe burial of those who die of EVD by trained workers with appropriate protective equipment. We describe the resources and response structure needed to implement such measures effectively, information that will assist in controlling future outbreaks.

## Introduction

Ebola Virus Disease (EVD) is a severe viral illness of humans, with recorded case fatality rates of up to 90% in past outbreaks [1]. The current EVD outbreak, caused by the Zaire strain of Ebolavirus, is the first recorded in West Africa. It commenced in Guinea in December 2013, then spread to neighbouring Liberia and Sierra Leone, and has exceeded all previous EVD outbreaks collectively in terms of number of cases and geographical distribution. Sierra Leone has reported the highest number of cases [2], with EVD overwhelming existing health services and response structures in many areas of the country [3]. Prior to the EVD outbreak, Sierra Leone already faced considerable challenges. A decade long major civil war from 1991–2002 has contributed to 40% of the population living in extreme poverty [4]. With 0·03 physicians per 1,000 population, its health system struggles to provide even basic services, resulting in high levels of morbidity and mortality due to endemic diseases such as malaria [5].

Transmission of EVD is primarily through close, unprotected contact with the body fluids of patients in the late stage of illness or following their death [6, 7]. These transmission characteristics facilitate and necessitate identification of contacts at risk of infection, their early admission to safe patient care facilities if illness occurs, and safe burial procedures for those that die. Such measures have successfully controlled EVD in the other settings [8–10], and are key to controlling the current outbreak. However, the structure and type of response activities leading to successful EVD control have not been well documented in the context of an outbreak of the scale and geographical distribution of the current outbreak in West Africa. The aim of this paper was to describe EVD transmission and the implementation of successful control measures in Kailahun District, Sierra Leone, and to consider implications for EVD control more broadly.

## Methods

Kailahun District (population of 350,000–450,000 [11] in an area of 4,859 km^2^) borders the region of Guinea in which the West African EVD outbreak commenced. Close ties between communities on both sides of this border facilitated the movement of people, and in this case, of disease. The first confirmed cases of EVD in the district were reported in late May 2014, and transmission peaked in August 2014 [12]. Following 42 continuous days without a confirmed case, Kailahun District was declared Ebola-free on 22 January 2015 [13].

We identified published and internal data from agencies involved in the EVD response in Kailahun district from June 2014 to June 2015. This included daily national situation reports and District-level data and reports of the Sierra Leone Ministry of Health and Sanitation, and Médecins Sans Frontières (MSF) patient data and internal epidemiological reports. We used EVD case definitions provided by the World Health Organisation over the course of the outbreak [2]. These data were used to map numbers of confirmed and probable EVD cases and response activities within Kailahun District. Data from the MSF Kailahun Ebola Management Centre (EMC) were analysed to assess epidemiological characteristics of confirmed EVD patients admitted to care. Characteristics included reported exposure (funeral-related or not), time interval between onset of illness and admission to the EMC, work-related exposures (health worker or not) and mortality. We then compared these characteristics between patients admitted during two time periods; June to July (the early period of response) compared with August to December (when coverage and quality of response had improved). This analysis relates to EVD transmission within Kailahun district. EVD patients were at times referred and brought by ambulance to Kailahun EMC from holding centres in other districts without adequate EMC capacity. Numbers admitted each day were dependant on EMC bed availability in Kailahun. These external referrals were excluded from this analysis. This study involved analysis of routinely collected surveillance and program data during the response to EVD in Kailahun district. Such analyses were conducted by program staff responsible for routine data management in response agencies, and results are presented in aggregate, anonymised form. This paper presents an analysis of routinely collected program data, an essential component of ongoing evaluation and quality improvement of clinical services in all settings. In keeping with international standards [14], in order to facilitate such essential quality assurance processes while at the same time safeguarding patient confidentiality, the MSF Ethics Review Board has determined requirements for exempting specific proposals addressing secondary analyses of routinely collected patient data from the requirement for formal human research ethical review [15, 16]. Specific written or verbal consent was therefore not sought from patients whose data contributed to this analysis. Data were analysed by staff responsible for entering and analysing data for routine patient care and program monitoring purposes and aggregate results are reported in this paper. Because of ethical restrictions, individual-level data cannot be shared publicly. The data are available under the terms of MSF's data sharing policy, found at http://fieldresearch.msf.org/msf/handle/10144/306501. Applications for access to MSF Datasets offered on the online catalogue should be submitted to MSF via data.sharing@msf.org.

We used mathematical modelling to evaluate the impact on EVD transmission of increased EMC capacity and improved case detection using a stochastic discrete-time model based around a local contact population (see full description in supporting material). As our aim was not to estimate parameters for Kailahun or to predict future case numbers, but to compare the broad effect of control measures, we have adopted a relatively simple model with a small number of parameters. The model is stochastic to allow for elimination of disease under successful control measures, and includes susceptible depletion within the local contact population. We consider the relative effect of increasing bed numbers under three surveillance response scenarios: Scenario A assumed surveillance activities detected 35% of EVD cases, Scenario B that 83% of cases were detected; the levels in these scenarios were based on analysis of program data (Table 1). Under Scenario C, we assumed 83% case detection, along with early admission and therefore less transmission from known contacts once symptomatic [7]. The model started with 100 cases, consistent with EVD case numbers in Kailahun over a generation of transmission (approximately 2–3 weeks) in June 2014. We assumed that patients not admitted to an EMC had a reproduction number of 1·7 [17], while patients that were admitted had a 50% reduction in their reproduction number to 0·85 [18]. Additionally, with earlier case detection and admission (Scenario C), we assumed the reproduction number decreased to 0·50 in secondary cases within each identified chain of transmission. We assumed an initial contact population for each case of 30 people and that 90% of transmission events occurred within this contact population. We conducted sensitivity analyses around all parameters to ensure that broad findings were not sensitive to these assumptions (see Supporting Material).

**Table 1:** Characteristics of confirmed cases resident in Kailahun District, and those admitted to Kailahun EMC, by period of outbreak (early stage of response (June to July 2014) vs comprehensive implementation of response (August to December 2014)).

	Total		Jun-Jul		Aug-Dec		p-value*
	N	%	N	%	N	%	p-value
Confirmed and probable EVD cases	652		388	-	264	-	
Confirmed EVD cases admitted to EMC (of all confirmed and probable EVD cases reported from in District)	354	63%	135	35%	219	83%	<0·001
Of confirmed EVD admissions							
Aged ≥5 years	332	94%	128	95%	204	93%	0·53
Female	174	49%	58	43%	116	53%	0·067
Health workers	18	5%	5	4%	13	6%	0·35
<3 days between symptom onset and admission ^	89	30%	22	19%	67	37%	<0·001
Reported funeral contact	79	22%	45	33%	34	16%	<0·001
Mortality	207	58%	81	60%	126	58%	0·72

* Comparing Jun-Jul with Aug-Dec;

^For the 299/354 (84%) of admitted cases with information on symptom onset.

## Results

**Fig 1:**
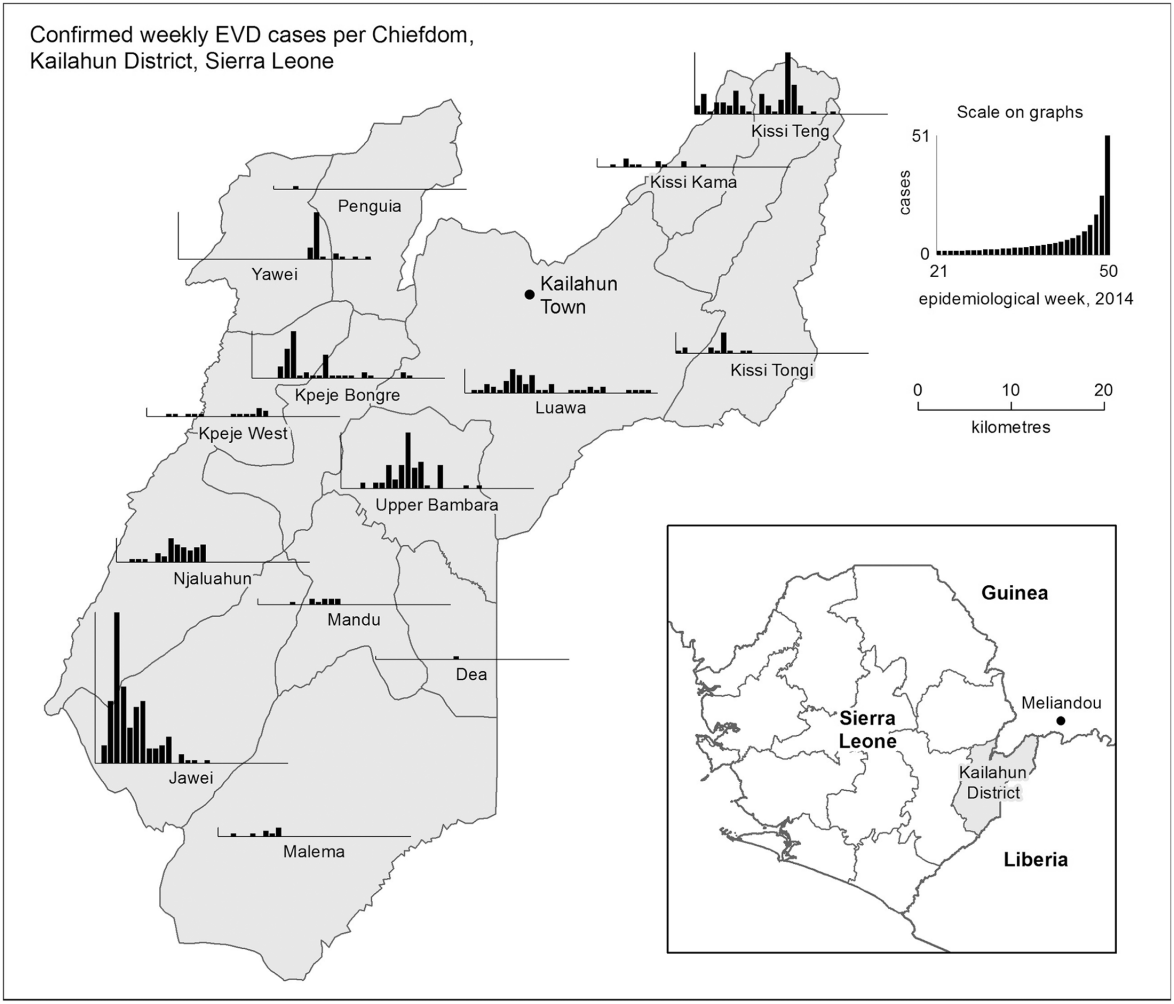
Weekly confirmed EVD cases per Chiefdom in Kailahun District, for the duration of the outbreak in the district. Also shown are the locations of the Ebola Management Centre (near Kailahun Town), and the epicentre of the outbreak in Guinea (Meliandou, inset map).

The chiefdom of Kissi Teng in Kailahun district closely borders the epicentre of the EVD outbreak in Guinea (Fig 1). EVD transmission was first identified in Kailahun in late May 2014, followed by a rapid increase in cases in June related to the funeral of a traditional healer in contact with EVD patients from Guinea [12]. 365 Ebola-related deaths have now been epidemiologically linked to this single funeral. The outbreak then spread from the Kissi Teng chiefdom to other chiefdoms throughout Kailahun district (Fig 1). The local Ministry of Health and Sanitation reported 652 EVD cases in district residents from June to December 2014, including 565 laboratory confirmed and 87 probable cases.

### Response measures

**Fig 2:**
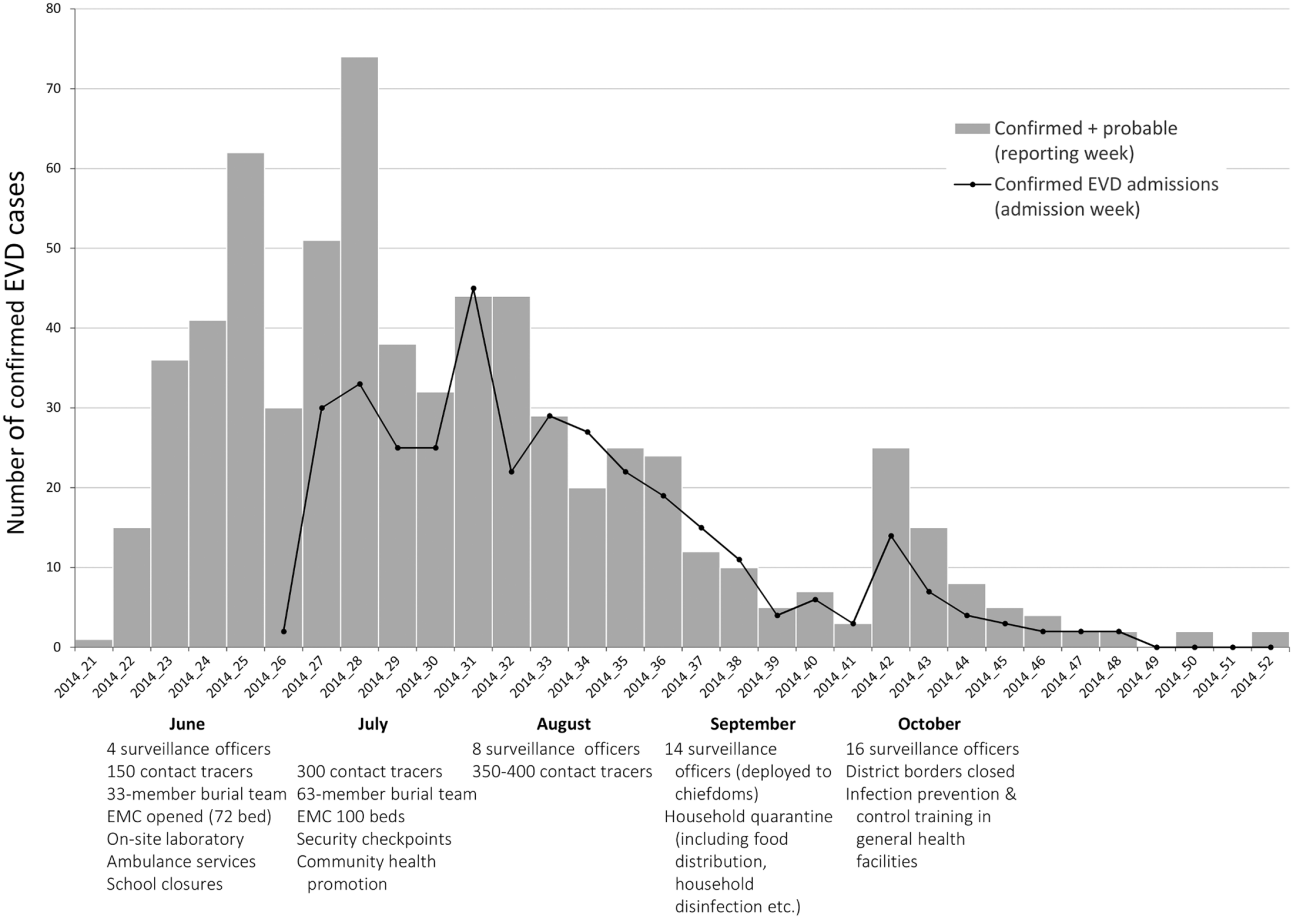
EVD case numbers (confirmed and probable by week of official reporting), confirmed EVD patients admitted to EMC (by date of admission) and key response activities in Kailahun district by epidemiological week. (Cases transferred to this EMC from outside Kailahun have been excluded from this summary.)

Response activities were initiated in May 2014, and early challenges included limited or no availability of most response activities, and community mistrust and fear affecting compliance with even the few available measures. By August 2014, safe patient care and burial services, surveillance, contact tracing and health promotion were in place and had increased in coverage (Fig 2). Contact tracers were community health workers (CHW) who prior to the EVD outbreak had been working with the District Health Management Team ensuring that essential services reach remote hard-to-reach communities where health centres are not present. They were selected by community stakeholders based on specific criteria, which included: resident in the community they represent; over 18 years of age; able to read and write; young and energetic; has interest in and willing to volunteer; and has moral standing and is well- respected in the community. A training package and standard operating procedures on contact tracing were developed by the Ministry of Health and Sanitation (MOHS) and training conducted based on these materials. Substantial increases in contact tracing staff and surveillance supervisors over time meant coverage of these measures increased from 1·0 surveillance personnel per confirmed case detected in June 2014 to 8·8 per confirmed case detected in September 2014. However, even with this increase, surveillance staff reported working long hours in order to ensure appropriate follow up of all reported cases and contacts. Quarantine measures restricting movement of household members of confirmed cases commenced in September 2014, and infection prevention and control activities in general health services commenced in October 2014.

The only EMC that operated in Kailahun opened on 26th June 2014. Information on the structure of clinical services within the EMC has been published previously [19]. From the outset all suspect, probable or confirmed cases could be referred to the EMC for epidemiological and clinical screening, laboratory testing and admission when warranted. Patients self-presented directly to the EMC, or were referred from general health services or from EVD holding facilities. From 36 beds in June 2014, EMC capacity expanded periodically, to 80 beds by early August and 100 by September in order to ensure that suspect, probable or confirmed cases of EVD were not refused or delayed admission once identified within the district. From September 2014 onwards laboratory results were generally available within 24 hours of sample collection for those admitted to the EMC.

Community-based health promotion activities constituted a core component of response. These health promotion activities were linked to patient care and surveillance through the EMC health promotion team. This team worked closely with patients and their families during admission to build understanding of the disease and trust in response measures. Accompanying patients who recovered and were discharged home was an important aspect of this team’s role, providing an opportunity to address stigma and enabling survivors to act as advocates within their community for early admission to care. The MSF health promotion team recruited and provided training and support to community health promotion volunteers, aiming to have one in each village in the district. These community health promotors (CHPs) were persons able to read and write in English and respected in the community. Their main role was to conduct health promotion activities in the village focussed on behaviour change related to risk factors for EVD transmission (caring for sick people and burial practices), prevention, health seeking behaviour and the services provided by the EMC. Specific practices in each village around these issues were also documented, and any rumours related to EVD that may influence community behaviour were addressed. CHP’s also reported cases and worked closely with contact tracers. This cooperation improved as the number of contact tracers increased.

CHP supervision and training was provided at primary healthcare unit (PHU) catchment area level with the support and agreement of the officer in charge of the PHU and village chiefs. The MSF health promotion team conducted CHP supervision and ongoing training, prioritising villages reporting cases for daily visits by both the EMC health promotion team and CHPs. Villages without current cases were visited either weekly or biweekly by supervisors and more frequently by CHPs. CHPs also met at PHU level weekly to discuss problems and solutions among themselves, and conducted discussions with community leaders, traditional healers and birth attendants, other groups and the general community. The number of CHP’s working in Kailahun increased to more than 600 by August 2014 and eventually to almost 1000 (one in each village).

### Coordination and management of response

Initially there was limited interaction at district level between response agencies, and between these agencies and affected communities. However, by September 2014 coordination across response agencies and health system levels was in place at district level, led by the surveillance staff of the MOHS District Health Management Team. Information exchange between district staff and community-level activities was directed through surveillance officers based in chiefdoms throughout the district. Surveillance staff also coordinated food and water distribution and hygiene activities in quarantined households assisted by contact tracing CHWs. Chiefdom leaders were members of the Ebola Taskforce. Local health facilities such as PHUs were also part of this network through their health officers.

### Patient characteristics

From its opening until January 2015, there were a total of 614 admissions to the EMC from Kailahun District, of which 354 (58%) tested positive for Ebola virus. The proportion of confirmed or probable cases of EVD detected in the district who were admitted to the EMC increased from 35% in June-July to 83% in August-December (p<0·001) (Table 1). The overall case fatality for EVD admissions was 58%, with no significant variation over time (case fatality was 60% in June-July and 58% in August-December 2014 (p = 0·72).

The proportion of confirmed cases from the District admitted to the EMC less than three days following onset of symptoms increased significantly (p<0·001), from 19% in June-July to 37% in August-December 2014 (Table 1). The proportion of confirmed cases reporting funeral contact decreased from 33% to 16% (p<0·001) over this period. Amongst Kailahun EMC admissions, 18 were health staff, constituting 4% of EVD cases reported in the district from June to July 2014, and 6% of those from August to December (p = 0·35). Exposures reported by health staff who were infected included work in general health facilities, work in the EMC, unpaid care of family members and relatives and community-based private health service provision. Individual health workers with EVD generally reported multiple exposures, both health facility and community-based.

### Mathematical modelling of response scenarios

Table 2 gives the results of mathematical transmission modelling to compare the relative effects of increased EMC capacity and increased surveillance under three scenarios, showing the mean number of cases in each generation, together with cumulative case numbers and the median reproduction number (R). Estimates are based on 5,000 simulations of the stochastic model, and 95% intervals about these estimates are provided in the Supplementary Material. Outbreak control is achieved when the median reproduction number declines to less than 1. We see that control cannot be achieved without sufficient bed capacity in the EMC: all three scenarios fail to reduce the reproduction number below 1 when there are 25 or 50 beds. However, increasing bed capacity alone is insufficient; under low levels of case detection and referral (Scenario A), increasing capacity from 75 to 100 beds has no effect on the outbreak because cases are not detected to fill these beds. Increasing the proportion of cases detected from 35% (Scenario A) to 83% (Scenario B) is required to bring the outbreak under control, once there are sufficient beds. Earlier isolation of epidemiologically linked cases through contact tracing (Scenario C) can further reduce case numbers. Sensitivity analyses around our choice of reproduction numbers confirmed the broad findings that both bed capacity and integrated surveillance are required to achieve control (see Supplementary Material). Findings under an analogous deterministic model were broadly similar to the mean of the stochastic model, although the deterministic model was unable to capture fade-out of disease.

**Table 2:** Cases per generation estimated using mathematical modelling, under varying numbers of beds in the Ebola Management Centre (EMC), and varying levels of surveillance and contact tracing. Scenario A: Surveillance activities detect 35% of cases in the community. Scenario B: Surveillance activities detect 83% of cases in the community. Scenario C: Surveillance activities detect 83% of cases in the community, and cases epidemiologically linked to a previously identified case are admitted earlier to the EMC.

EMC bed capacity	Control scenario	New cases per generation of transmission				Cumulative cases	median R*
		0	1	2	3		
25	Scenario A	100	144	206	290	740	1·42
	Scenario B	100	144	206	289	739	1·42
	Scenario C	100	144	196	239	679	1·31
50	Scenario A	100	135	177	229	641	1·31
	Scenario B	100	123	155	197	575	1·26
	Scenario C	100	124	141	143	508	1·12
75	Scenario A	100	136	176	219	631	1·29
	Scenario B	100	103	104	104	411	1·01
	Scenario C	100	103	85	53	341	0·84
100	Scenario A	100	136	177	220	633	1·29
	Scenario B	100	96	90	82	368	0·94
	Scenario C	100	96	69	41	306	0·78

* Median reproduction number over generations 0 to 3.

## Discussion

During the 2014–15 Ebola epidemic, a comprehensive response against Ebola was established in Kailahun district, Sierra Leone. The majority of EVD cases detected in the district were admitted to the EMC, and this proportion increased significantly over time. There was also a statistically significant increase in the proportion of patients admitted within 3 days of disease onset and in the proportion reporting funeral contact compared to the early period of the outbreak response. This suggests that once a comprehensive response was in place, the majority of EVD patients resident in the district were identified, safely cared for when symptomatic through admission to an EMC, and if they died, received a safe burial; thus decreasing the risk of EVD transmission to their family and community. A recent paper has published similar findings in relation to reduction in funeral attendance [20]. Our findings build on this by identifying interventions associated with such improvements in epidemiological risk factors for transmission.

Although challenging to implement, adequate EMC bed capacity, as was called for early in the West African outbreak [21], is vital to EVD control. This capacity must be available promptly, and must be accessible to patients with suspect, probable or confirmed EVD in order for early case detection to have an impact on transmission in the community and general health services through safe care of those who are ill. Once established, rapid availability of laboratory diagnostic results supported appropriate patient management and contact tracing activities.

A second key finding is that surveillance and contact tracing are essential to increasing numbers admitted to care and in reducing delay to admission. We found that 8·8 personnel per confirmed case, while imposing a heavy workload on staff, achieved adequate surveillance and contact tracing coverage in a rural, geographically dispersed population where individuals were known to each other at community level. Applicability of this coverage level in urban areas would be dependent on population density, catchment size and social connections within the community.

The value of the mathematical modelling is that it allows us to assess the effect of increased bed numbers and improved surveillance separately, while our data (Fig 2) reflects a simultaneous increase in bed numbers and epidemiologists conducting contact tracing. Nevertheless, our modelling finding that contact tracing is needed in addition to bed numbers is supported by the trend in case numbers, which began to decline in August following an increase in contact tracing.

Community engagement is crucial if surveillance and contact tracing activities are to achieve high levels of early case detection and appropriate management [22]. Unless communities understand the value of response measures, lack of awareness and fear will prevent compliance. In Kailahun, health promotion staff worked with community leaders, with patients and their families, with survivors, and with surveillance and contact tracing staff to build trust and convey appropriate messages around EVD control. Increased community awareness and understanding is likely to have been important in improving early admission to care and reduced funeral contact. Health promotion activities also covered other behaviour modification messages such as avoiding contact with the sick and regular handwashing, and this may also have contributed to decrease in community transmission.

The clearly defined catchment zone of Kailahun district was of a size that allowed it to function as a single health service area, enabling close links between individuals involved in response activities within and across different health system and administrative levels and with communities. These links enabled treatment, detection, and prevention activities to be integrated into a combined response strategy. This may be one factor which contributed to successful control in Kailahun, but is challenging to replicate in other settings such as large urban centres. The capital of Sierra Leone, Freetown, is such a setting. The Freetown area reported its first case of confirmed EVD on 23 June 2014, and case numbers peaked at 200–250 confirmed EVD cases per week in November 2014 [23]. Cases in this area began decreasing in December 2014, associated with an increase in EMC capacity, but contact tracing and case detection remained poor [12]. A program commenced in January 2015 in Freetown to support community-level response activities such as surveillance and contact tracing, with specific agencies each responsible for supporting a defined zone, and there was a further decrease in transmission from January 2015 onwards following implementation of these measures [23]. Based on our findings, we support this as a promising strategy for replicating the structure of response in Kailahun within a much larger urban setting.

Mathematical modelling of intervention scenarios assessed the importance of both EMC bed capacity and surveillance and control activities to detect cases early in their infectious period. There has been considerable modelling work done to predict future Ebola case numbers [17, 24, 25]. The aim of the modelling work presented here was to assess the relative importance of these components of Ebola response. The results were highly robust to model assumptions, and confirmed the epidemiological findings of this paper that both components are needed to control EVD.

Mortality for those admitted to the EMC remained similar throughout the outbreak in Kailahun. Data from past outbreaks and from other areas of Sierra Leone show that the average time from disease onset to death is 4 days, and the majority of those who die do so within the first 10 days of disease onset [17, 26]. As response improved, the proportion of patients admitted within 3 days of symptom onset to the Kailahun EMC almost doubled. As mortality is highest in the first week of illness, we expect that an increase in the proportion of patients admitted early in their illness would be associated with increased overall mortality in those admitted to the EMC. Such an increase may have hidden any reduction in mortality in EMC patients due to factors such as earlier access to or improvements in care.

Health workers comprised 5·1% of EVD cases detected in Kailahun. This is consistent with findings from the country as a whole, with 5·2% of cases reported up to November 2014 in health workers [27]. Despite an overall decrease in case numbers over these periods, the proportion of cases who were health care workers did not decrease over time. It indicates that health staff continued to be at risk of EVD even in the latter stages of the outbreak. Health workers reported a range of health facility and community-based exposures. There was also limited focus in Kailahun on infection prevention and control activities in general health facilities until late in the outbreak, with the first training for health staff held in the district in October 2014. Even at that time, there continued to be very limited availability of chlorine, gloves, masks and water at the level of PHUs. This was also the case in Sierra Leone more generally [28, 29]. Delays in implementing infection control in general health services are likely to have contributed to health worker infections in Kailahun, and must be a focus of current and future EVD response for general health facilities as well as EMC’s.

It is clear from our results that all components of response are important and must be funded appropriately in order to ensure timely and adequate coverage of affected areas. Our findings suggest that in Kailahun there were delays in the implementation and scale-up of control activities such as recruitment of adequate contact tracing and health promotion staff and infection prevention and control in general health services. Recent commentary has identified delays in the availability and disbursement of funding during this current outbreak [30]. The contribution of such delays to the scale of the outbreak is an important area for future research and analysis.

Limitations to this study include that we based our conclusions on cases detected and response activities within a single district. Although we believe that the response measures implemented in Kailahun contributed to control of EVD transmission, there may have been other contributing factors we are unaware of. Data on epidemiological characteristics such as date of onset and type of exposure were from patients confirmed with EVD in the Kailahun EMC facility, and therefore may not reflect the situation in the general community. The quality of data collection is likely to have improved over time as training and staffing increased, particularly for data from surveillance and contact tracing activities. Any bias due to improved case detection would result in an apparent increase in cases over time, suggesting the decrease in cases seen is a reliable finding. Data from the EMC also varied in quality. Variables such as age and sex were recorded consistently. However, completion of data on variables such as reported date of onset and source of exposure varied over time, with no consistent pattern. These variables were possibly affected by staff workload, but this affected all admissions during a particular period and therefore is unlikely to result in bias. Data on behaviour and contact history were based on patient report, and it is possible that public messaging encouraging early presentation and avoiding funeral contact may have influenced reporting rather than actual behaviour. Our mathematical model was designed for a small population centre where most transmission occurs within local population groups; transmission patterns likely differ somewhat in larger population centres such as Freetown.

Conclusion: Our study describes EVD transmission and the implementation of successful control in a rural district of Sierra Leone in a period when other areas of the country and the region were experiencing high case numbers and limited control. The key factor in achieving control appears to be admission of the majority of EVD cases to an appropriate care facility early in their illness. This requires sufficient EMC bed capacity (including the capacity to admit both suspect and probable cases for assessment) integrated with adequate community-based case detection and prevention activities. Information we present on the structure, type and level of intervention associated with achieving control in Kailahun may inform effective EVD response in future outbreaks.

## Supporting Information

S1 File Supplementary material providing further details of the mathematical model. (DOCX)
