# Supplementary material for: Successful Control of Ebola Virus Disease: Analysis of Service Based Data from Rural Sierra Leone
Source: PLoS Negl Trop Dis. 2016 Mar 9;10(3):e0004498. doi: 10.1371/journal.pntd.0004498 (PMC4784943; doi:10.1371/journal.pntd.0004498)
Supplement: S1 File — (DOCX) [file pntd.0004498.s001.docx]

# Supplementary Material for “Successful control of Ebola Virus Disease: analysis of service based data from rural Sierra Leone”

This supplementary material provides further details of the mathematical model.

Model structure

We developed a simple stochastic discrete-time model with time-step equal to the serial interval for Ebola. Given the importance of transmission of Ebola within family groups, we allowed for most transmission to occur within a local contact population, together with some transmission outside these populations. Figure S1 provides a schematic diagram of this model, where each box represents a local population and we compare the situation for controlled and uncontrolled Ebola cases. While the local contact population is likely to be based around households, it also includes extended family members. We assumed that a proportion *p* of contacts occur within this population, and modelled local susceptible depletion. The number of people infected by a case was assumed to depend on control measures, with *R_u_* people infected by a case that is not controlled, *R_cp_* people infected by a case that is controlled and is the first controlled case in the local population, and *R_cs_* people infected by a case that is controlled and is a secondary case controlled in that local population.

Interventions assessed:

Over June-December 2014, EMC capacity in Kailahun increased to 100 beds, while the proportion of confirmed cases admitted to the EMC increased from 35% to 83%. We quantify the importance of both bed capacity and case detection in the model by comparing case numbers under bed capacity of 25, 50, 75 and 100, and with three case detection scenarios:

*Scenario A*: Surveillance activities detect 35% of cases in the community

*Scenario B*: Surveillance activities detect 83% of cases in the community

*Scenario C*: Surveillance activities detect 83% of cases in the community and cases in
the same local population as a previously identified case are admitted earlier to the EMC

For all three scenarios, we assume that the reproduction number of controlled cases (*R_cp_* and *R_cs_*) is less than that for uncontrolled cases (*R_u_*). In scenarios A and B, we assume the reproduction number of the first detected case in a local population (*R_cp_*) is the same as that for subsequently detected cases (*R_cs_*), however in scenario C we assume *R_cs_* is less than *R_cp_.*

Figure S1: Model diagram for a discrete-time stochastic model with a mix of controlled and uncontrolled cases. Grey boxes indicate local contact populations in the model, and arrows indicate transmission events, with a proportion *p* of events occurring within the local population, and a proportion *1-p* of infection events occurring outside the contact population. A case that is not controlled infects *R_u_* other cases on average, while a case that is not controlled infects *R_cp_* other cases if it is the first case detected in that local population, and *R_cs_* other cases if it a secondary detected case in that population.

(1-p)R_cp_

pR_cp_

(1-p)R_cs_

pR_cs_

Controlled cases

(1-p)R_u_

pR_u_

Uncontrolled cases

Parameter estimates:

Our assumptions around bed capacity and the proportion of cases detected rely on data from Kailahun. As these data largely arise from a time when Ebola was established and we did not have full infection chains for the outbreak, we did not feel it was appropriate to attempt to estimate reproduction numbers, but instead used estimates from the literature, together with sensitivity analyses around these estimates. As our dataset was not large and included control measures we have not based our choice of reproduction numbers on our data, and have instead used estimates from larger Ebola datasets. In our base model presented in the paper, we assumed that patients not admitted to an EMC (“uncontrolled cases”) had a reproduction number *R_u_*=1.7^1^, while patients admitted to an EMC (“controlled cases”) had a reproduction number *R_cp_*= 0.85^2^. Where follow-up of patients allowed earlier detection of cases (scenario C), we assumed that the reproduction number of secondary detected cases, *R_cs_* =0.5. In our base model, we assumed a local contact population of 30, and that *p*=0.9. These estimates are consistent with our data; with a generation interval of two weeks, case numbers for June and early July indicate a reproduction number lying between 1.4 and 2.1, while control measures have clearly brought the reproduction number below one towards the end of the time period (Figure 2).

Initial conditions and sensitivity analyses:

As we are interested in the effect of control measures once disease is established, we started the model with 100 cases, consistent with Ebola numbers in Kailahun over a generation of infection in June 2014.

As our aim with this model is to assess the relative effects of control measures, rather than to quantify or predict exact number of Ebola cases, we tested the broad findings of the model under a range of values for the reproduction numbers, including values for *R*_u_ between 1.3 and 2.1, R_cp_ between 0.5 and 0.9, local contact populations ranging from 10 to 50, and *p* between 0.8 and 0.95. While numbers of Ebola cases differed under these assumptions, the broad finding that both EMC capacity and surveillance numbers were needed to ensure control of Ebola was robust across these model assumptions.

Finally, while we chose to use a stochastic model to ensure integer numbers of infected individuals over time, a comparison of the model with a deterministic analogue indicated that the mean values of the stochastic model were consistent with a deterministic version of the model. For clarity, we have not included intervals about the cases per generation presented in Table 2 of the main text. Table S1 includes the intervals that contain 95% of simulated values for each generation.

References

^1^ WHO Ebola Response Team. Ebola virus disease in West Africa--the first 9 months of the epidemic and forward projections. *N Engl J Med* 2014; **371**(16): 1481-95.

^2^ Faye O, Boelle PY, Heleze E, et al. Chains of transmission and control of Ebola virus disease in Conakry, Guinea, in 2014: an observational study. Lancet Infect Dis 2015; **15**(3): 320-326.

**Table S2**: Cases per generation with 95% intervals estimated using a discrete-time stochastic mathematical model under varying numbers of beds in the Ebola Management Centre (EMC), and varying levels of surveillance and contact tracing. Estimates are based on 5,000 runs of the model.

Scenario A: Surveillance activities detect 35% of cases in the community.

Scenario B: Surveillance activities detect 83% of cases in the community.

Scenario C: Surveillance activities detect 83% of cases in the community, and cases epidemiologically linked to a previously identified case are admitted earlier to the EMC.

| EMC bed capacity | Control scenario | New cases per generation of transmission | | | |
| --- | --- | --- | --- | --- | --- |
|  |  | 0 | 1 | 2 | 3 |
| 25 | Scenario A | 100 | 144 (120, 169) | 206 (162, 254) | 290 (221, 366) |
|  | Scenario B | 100 | 144 (121, 167) | 206 (161, 254) | 289 (218, 365) |
|  | Scenario C | 100 | 144 (122, 168) | 196 (153,242) | 239 (178, 305) |
| 50 | Scenario A | 100 | 135 (113, 158) | 177 (139, 218) | 229 (169, 295) |
|  | Scenario B | 100 | 123 (102, 145) | 155 (115, 197) | 197 (134, 265) |
|  | Scenario C | 100 | 124 (103, 147) | 141 (105, 182) | 143 (91, 200) |
| 75 | Scenario A | 100 | 136 (113, 159) | 176 (139, 216) | 219 (168, 274) |
|  | Scenario B | 100 | 103 (83, 123) | 104 (71, 142) | 104 (61, 163) |
|  | Scenario C | 100 | 103 (84, 123) | 85 (55, 121) | 53 (29, 94) |
| 100 | Scenario A | 100 | 136 (113, 159) | 177 (139, 216) | 220 (167, 274) |
|  | Scenario B | 100 | 96 (77, 116) | 90 (66, 116) | 82 (61, 163) |
|  | Scenario C | 100 | 96 (77, 116) | 69 (49, 91) | 41 (25, 59) |
